# Immunological insights into COVID-19 in Southern Nigeria

**DOI:** 10.3389/fimmu.2024.1305586

**Published:** 2024-01-23

**Authors:** Chinedu A. Ugwu, Oluwasina Alao, Oluwagboadurami G. John, Blossom Akinnawo, Israel Ajayi, Ooreofe Odebode, Ifeoluwa Bejide, Allan Campbell, Julian Campbell, Jolly A. Adole, Idowu B. Olawoye, Kazeem Akano, Johnson Okolie, Philomena Eromon, Peter Olaitan, Ajibola Olagunoye, Ibukun Adebayo, Victor Adebayo, Elizabeth Babalola, Omowumi Abioye, Nnennaya Ajayi, Emeka Ogah, Kingsley Ukwaja, Sylvanus Okoro, Ogbonnaya Oje, Ojide Chiedozie Kingsley, Matthew Eke, Venatius Onyia, Olivia Achonduh-Atijegbe, Friday Elechi Ewah, Mary Obasi, Violet Igwe, Olufemi Ayodeji, Abejegah Chukwuyem, Sampson Owhin, Nicholas Oyejide, Sylvester Abah, Winifred Ingbian, Moyosoore Osoba, Ahmed Alebiosu, Angalee Nadesalingam, Ernest T. Aguinam, George Carnell, Nina Krause, Andrew Chan, Charlotte George, Rebecca Kinsley, Paul Tonks, Nigel Temperton, Jonathan Heeney, Christian Happi

**Affiliations:** ^1^ The Africa Centre of Excellence for Genomics of Infectious Diseases (ACEGID), Redeemer’s University, Ede, Osun, Nigeria; ^2^ Department of Biological Sciences, Faculty of Natural Sciences, Redeemer’s University, Ede, Osun, Nigeria; ^3^ Osun State University Teaching Hospital (UNIOSUNTH), Osogbo, Nigeria; ^4^ Alex Ekwueme Federal University Teaching Hospital Abakaliki (AEFUTHA), Abakaliki, Nigeria; ^5^ Federal Medical Centre (FMC), Owo, Nigeria; ^6^ Laboratory of Viral Zoonotics, Department of Veterinary Medicine, University of Cambridge, Cambridge, United Kingdom; ^7^ Viral Pseudotype Unit, Medway School of Pharmacy, The Universities of Greenwich and Kent, Kent, United Kingdom

**Keywords:** COVID-19, SARS-CoV-2, immunity, vaccine, Nigeria, pre-pandemic, preexisting

## Abstract

**Introduction:**

One of the unexpected outcomes of the COVID-19 pandemic was the relatively low levels of morbidity and mortality in Africa compared to the rest of the world. Nigeria, Africa's most populous nation, accounted for less than 0.01% of the global COVID-19 fatalities. The factors responsible for Nigeria's relatively low loss of life due to COVID-19 are unknown. Also, the correlates of protective immunity to SARS-CoV-2 and the impact of pre-existing immunity on the outcome of the COVID-19 pandemic in Africa are yet to be elucidated. Here, we evaluated the natural and vaccine-induced immune responses from vaccinated, non-vaccinated and convalescent individuals in Southern Nigeria throughout the three waves of the COVID-19 pandemic in Nigeria. We also examined the pre-existing immune responses to SARS-CoV-2 from samples collected prior to the COVID-19 pandemic.

**Methods:**

We used spike RBD and N- IgG antibody ELISA to measure binding antibody responses, SARS-CoV-2 pseudotype assay protocol expressing the spike protein of different variants (D614G, Delta, Beta, Omicron BA1) to measure neutralizing antibody responses and nucleoprotein (N) and spike (S1, S2) direct ex vivo interferon gamma (IFNγ) T cell ELISpot to measure T cell responses.

**Result:**

Our study demonstrated a similar magnitude of both binding (N-IgG (74% and 62%), S-RBD IgG (70% and 53%) and neutralizing (D614G (49% and 29%), Delta (56% and 47%), Beta (48% and 24%), Omicron BA1 (41% and 21%)) antibody responses from symptomatic and asymptomatic survivors in Nigeria. A similar magnitude was also seen among vaccinated participants. Interestingly, we revealed the presence of preexisting binding antibodies (N-IgG (60%) and S-RBD IgG (44%)) but no neutralizing antibodies from samples collected prior to the pandemic.

**Discussion:**

These findings revealed that both vaccinated, non-vaccinated and convalescent individuals in Southern Nigeria make similar magnitude of both binding and cross-reactive neutralizing antibody responses. It supported the presence of preexisting binding antibody responses among some Nigerians prior to the COVID-19 pandemic. Lastly, hybrid immunity and heterologous vaccine boosting induced the strongest binding and broadly neutralizing antibody responses compared to vaccine or infection-acquired immunity alone.

## Introduction

Given the pace of SARS-CoV-2 transmission, its relatively high morbidity and mortality rate, and its global impact, COVID-19 has recently become one of the most severe pandemics. Over six hundred and ninety million people were infected, and almost seven million died globally (1). This virus’s indiscriminate and rapid spread across international borders resulted in mild, moderate, and severe outcomes, necessitating a variety of public health responses in different countries and among different demographics. Nigeria, the most populated country in Africa with many highly populated cities and fragile health care, is poised for an explosive spread of SARS-CoV-2. Interestingly, as of October 2023, Nigeria’s reported confirmed infections (266675 cases) and mortality in only (3155 individuals), which were significantly lower than other highly populated countries (1). The reasons for this are not entirely clear, but certain factors, such as the mixing of the population (increased exposure) and the immunological status of its population, may be responsible for the differences in the outbreak in Nigeria (2). With the availability of vaccines, the global morbidity and mortality from COVID-19 has been greatly reduced. Efforts are directed towards understanding the features of natural, vaccine-induced and combined (hybrid) protective immunity that will provide greater insight and understanding to increase preparedness, prevent or contain future pandemics (3, 4). Studies have shown that the humoral (neutralizing antibody) responses are important in blocking the entry of SARS-CoV-2 and reducing fatal COVID-19 diseases, but lower titres do not prevent SARS-CoV-2 infection (5). With numerous variants of concern capable of evading the antibody (neutralizing) response, the T-cell response has been shown to be an important second barrier to disease and more durable (5–7). Moreso, combined or hybrid immunity, the acquisition of both vaccine and naturally acquired immunity through exposure and infection, has been documented to induce the most robust immune responses and provided the greatest cross-protection against the different variants of SARS-CoV-2 (8). Preexisting immunity from seasonal coronavirus may potentially result in cross-protection against SARS-CoV-2 in different regions of the world (9). It has also been proposed that immunity from burden of concurrent exposure to other diseases has been responsible for the less catastrophic outbreak in Africa compared to other parts of the world. However, the impact of both pre-existing and community acquired immunity during the pandemic is yet to be elucidated in Nigeria. Notably, the vast majority of SARS-CoV-2 immune correlates data from the COVID-19 pandemic are based on reports from developed nations, with a paucity of immunological data emanating from Africa where the pandemic was less catastrophic than in other parts of the world (10).

This study assessed both the natural and vaccine-induced immune responses from vaccinated, non-vaccinated and convalescent individuals in Southern Nigeria throughout the three waves of the pandemic in Nigeria. Also, we assessed pre-existing immune response from patient’s samples collected prior to the COVID-19 pandemic. Our data provides immune correlate data from Africa’s most populous nation and measures the impact of pre-existing immunity on the outcome of the COVID-19 pandemic.

## Methods

### Study participants

The study participants were enrolled through ACEGID Clinical site networks in Abakaliki (Alex Ekwueme Federal University Teaching Hospital Abakaliki (AE-FUTHA), Ebonyi State), Owo (Federal Medical Centre (FMC) Owo, Ondo State), Osun State University Teaching Hospital (UNIOSUNTH) Osogbo, Osun State, Nigeria between February 2021 and December 2022. The study comprised four categories of participants: (i) hospitalized and COVID-19 convalescent patients (survivors N=89) with a negative SARS-CoV-2 polymerase chain reaction [PCR]/RDT tests at the time of sample collection, (ii) exposed non hospitalized asymptomatic contacts (exposed asymptomatic participants N=34), (iii) SARS-CoV-2 vaccinees (N=517) and (iv) prepandemic sera (N=64) (**Tables 1**, **2**). The inclusion criteria is that the participant is able and willing to provide written consent or assent (if underage) to participate in the study and willing to share contact and location for follow up study and for the vaccinated cohorts, must have received a single or complete dose(s) of either the AstraZeneca (AZD-1223), or Janssen (Ad26.CoV2.S) vaccine, Pfizer or Moderna COVID-19 vaccines administered preferably within three months but not more than six months prior to study enrollment. The exclusion criteria on the other hand, is that the participation do not have a positive or confirmed SARS-CoV-2 polymerase chain reaction [PCR]/RDT tests at the time of sample collection. Also, that the participant do not have any significant condition (medical, psychological, psychiatric, or social), which, in the judgment of the study investigator, might interfere with the conduct of the study. There were more females (60%, 62%) than males (40%, 38%) in both COVID-19 survivors and vaccinees. The mean age for both survivors and the vaccinees were 36.03(± 14.89 years) and 38.09(± 10.76 years) at 95% confident interval respectively.

**Table 1:** The seroprevalence of SARS-CoV-2 S-RBD and N binding (IgG) and neutralizing antibody response among survivors and their contacts in Southern Nigeria.

	ELISA			Virus Pseudotype Neutralization		
Subpopulation	N-IgG (%)	S-RBD IgG (%)	D614G (%)	Beta (%)	Delta (%)	Omicron BA1(%)
Survivor	74	70	49	48	56	41
Contact	62	53	29	24	47	21
Pre-pandemic	60	44	0	0	0	0

**Table 2:** Vaccine and convalescent sera distribution.

Vaccines	Doses	Negative to SRBD and N	Positive to N	Positive to SRBD	Positive to SRBD and N	Total
AstraZeneca	Single	10	4	21	24	59
	Double	2	3	17	45	67
	Third			1		1
Pfizer	Single	7	7	14	41	69
	Double	1	5	15	43	64
	Third		2	15	40	57
Moderna	Single	6	7	10	29	52
	Double	4	7	24	39	74
	Third				1	1
Janssen	Single	6	1	14	24	45
	Double	3	2	8	15	28
	Third					
		39	38	139	301	517
%Total		7.54	7.35	26.89	58.22	

### PBMC isolation and serum separation

Plasma and peripheral blood mononuclear cells (PBMCs) were separated immediately following manufacturer instructions (Sigma‐Aldrich, Z642843). 10mL of whole blood was transferred from the EDTA tubes into LeucoSep-tube containing ficoll-hypaque at a ratio of 2:1. The tube was centrifuged at 800 x g for 30 minutes at room temperature in a swinging-bucket rotor with no break. The top layer of plasma was removed, and the buffy coat interface was collected, washed twice with PBS-EDTA (10 mM), and centrifuged for 10 minutes at 250 x g with the brake on. The pelleted cells were suspended in red blood cell lysis buffer (1 mM KHCO3, 0.15 M NH4Cl, 0.1 mM EDTA, HCl pH 7.2 to 7.4) at room temperature for 5 minutes. The cells were washed again with PBS-EDTA, centrifuged at 250 x g for 10 minutes at 4°C and resuspended in appropriate medium (Leibovitz medium, Sigma-Aldrich, L1518) for further assay (ELISpot). The plasma was centrifuged at 250 x g for 5 minutes at 4°C and transferred to a new 15 mL tube to remove cells and debris. Both the PBMCs and plasma were transferred to 2mL cryotubes for further assay (ELISpot and ELISA) and storage at -80°C.

### ELISpot

PBMCs were re-suspended in 10 mL of media (500 mL Leibovitz media supplemented: 5 mL Pen/Strep, 5 mL L-glutamine, 12.5 mL HEPES, 0.5 mL 2-mercaptoethanol) and were plated onto customized ELISpot plates (Catalogue no: 10602KMM) coated with IFNγ (2x10^5^ cells/well) according to manufacturer’s instructions. 100µl (1µg/mL) of PepMixTM SARS-CoV-2 spike peptides (JPT, PM-WCPV-S-1 (pooled into S1 and S2 covering the entire SARS-CoV-2 spike) and the nucleoprotein peptides (JPT, PM-WCPV-NCAP1) were added to each well according to the plate map (see **Supplementary Table 1**). All peptides were 15mer with 11 amino acid overlaps. anti CD3 and vehicle control (media) was then added separately to individual wells of the customized ELISpot plates containing the PBMC (anti CD3 and vehicle control (media) were used as both positive and negative controls respectively). The plate was incubated in the hood for 20-24 hours at 37°C and 5% CO_2_ with no disturbance. After incubation, 80µL of detection solution was added to each well and incubated for 2 hours at room temperature following washing twice with 0.05% Tween-PBS. Thereafter, the detection solution was decanted and wells were washed three times with 0.05% Tween-PBS and incubated with 80uL of tertiary solution for another 30 minutes at room temperature. The plate was later washed two times with 0.05% Tween-PBS and two times with distilled water, 200µL/well each time. 80uL/well of blue developer solution was added and incubated for 15 minutes at room temperature. The reaction was stopped by gently rinsing the membrane with tap water, and decanting; this step was repeated three times. The protective underdrain was removed, and the back of the plate was also rinsed with tap water. The plate was air-dried for 24 hours face down on paper towels on the bench top. Scanning and plate count was done using CTL immunospot counter. For all wells, the numbers of spot forming units (SFU) were determined using SmartCountTM and Autogate. Tests and controls were carried out in duplicates for each sample. Counts per sample were obtained by subtracting the mean of background SFU (Media) from the mean of peptide specific SFU then expressed as SFU/million PBMCs. The threshold for detection of a positive response was assigned 40 SFU/million PBMCs; this is the mean SFU multiplied by 3x standard deviation from three known negative samples.

### ELISA

ELISA was performed on human plasma using ReSARSCoV-2 (S-RBD) and ReSARSCoV-2 N IgG ELISA Test Kit (10180 and 10166, Zalgen Labs, LLC) with either S-RBD or N as the capture antigens according to the manufacturer’s instructions. Lyophilized human monoclonal calibrator and negative control plasma were reconstituted with 0.10 mL and 0.25 mL laboratory-grade water respectively. Calibrator was diluted 1:101 (0.01 mL/1.0 mL followed by four threefold serial dilutions to create a calibration curve for antibody concentration estimation. Calibrator (or Reference) dilutions, diluted negative control and patient samples (1:100) were transferred (0.1 mL/well) in duplicate wells. Microwell plates were incubated at ambient temperature (18–30°C) for 30 minutes. Microwell plates were washed four times with 0.05% Tween-PBS wash buffer. Anti-human IgG or IgM-horse- radish peroxidase conjugated reagent was added to each well (0.1 mL/well) followed by a 30 minute incubation at ambient temperature. After repeating the PBS-Tween wash, 3,3′,5,5′-Tetramethylbenzidine (TMB) Substrate was added to each well (0.1mL/well). The TMB substrate was incubated for 10 minutes followed by the addition (0.1 mL/well) of Stopping Solution (2% Methane sulfonic Acid). Developed ELISA plates were read at 450 nm (with 650 nm reference). IgG concentration was estimated using the Optical Density (OD) reading from the ELISA plate reader. The negative cut-off (0.3) was determined as the mean multiplied by three standard deviations of three known negative samples (mean(3SD) three samples from participants with no prior exposure to SARS-CoV-2).

#### Virus Pseudotype Neutralization Assay

We used SARS-CoV-2 pseudotype assay protocol described by Di Genova et al. (2020) (11). To produce the pseudotyped Viruses (PVs) expressing the spike protein of different variants (D614G, Delta, Beta, Omicron BA1), HEK293T/17 cells were transfected with HIV Gag-pol, pCSFLW firefly luciferase and the SARS-CoV-2 spike plasmids using FuGENE-HD, incubated for 48 hours at 37°C and 5% CO2, and the supernatant harvested. To determine the titre of the PVs, on day 1 the HEK293T/17 cells were transfected with ACE-2 and TMPRSS2 plasmids to be used as target cells. On day 2, on a 96-well plate, the PV supernatants were serially diluted 1:2 in DMEM. The target cells were added to the 96-well plate at 10,000 cells/well. The PV production titre (in relative light units per ml; RLU/ml) was calculated from the luciferase expression measured on day 4 using Bright-Glo (Promega) reagent with the luminometer GloMax Explorer (Promega). The neutralising IC_50_ of the human sera was determined by serially diluting the samples 1:5 in DMEM in a 96-well plate and incubating for one hour with 5x10^5^ – 5x10^6^ RLU per well of PV. Transfected target cells (as above) were added at a density of 10,000 cells per 96-well and incubated for 48 hours. The RLU was measured as above, and the IC_50_ calculated using GraphPad Prism according to Ferrara and Temperton (2018) (12).

#### Data analysis and statistical methods

Data was analyzed using Microsoft Excel (version 16.39, Microsoft, Redmond, WA) and GraphPad Prism (version 8.4.2, 2020, GraphPad Software, Inc., San Diego, CA). Discrete and categorical variables were presented as frequencies and percentages and were compared using test of proportion by calculating chi-square. Continuous variables were presented as geometric mean (GM) and 95% confidence interval of the GM and compared using a non-parametric test (Mann-Whitney U test). All tests of significance were two-tailed and values of P < 0.05 were indicative of statistical significance.

#### Ethical approval

All methods were carried out in accordance with relevant guidelines and regulations. All subjects enrolled in this study and/or their legal guardians provided written informed consent. Human subjects testing and sample collection, was approved by the Redeemer’s University Institutional Review Board, the Nigerian National Health Research Ethics Committee (SIP-NG-NHREC/01/01/2007-12/01/2021, ARISE-NHREC/01/01/2007-11/02/2022), Federal Medical centre (FMC), Owo, Alex Ekwueme Federal Teaching Hospital (AE-FUTHA) Ethics and Scientific Research Committee and the University of Cambridge Institutional Review Board. Once informed consent is obtained from the participants, blood samples were collected from study participants and processed in the Virology Laboratory at the Alex Ekwueme Federal University Teaching Hospital (AE-FUTHA) Abakaliki Ebonyi State and Federal Medical Centre (FMC) Owo, Ondo State. Only qualified Nigerian medical personnel and laboratory staff were involved in the administration of questionnaire and sample collection from the participants.

## Results

### Both hospitalized convalescent and non-hospitalized exposed contacts make similar binding and neutralizing antibody responses to SARS-CoV-2 antigens.

**Figure 1:**
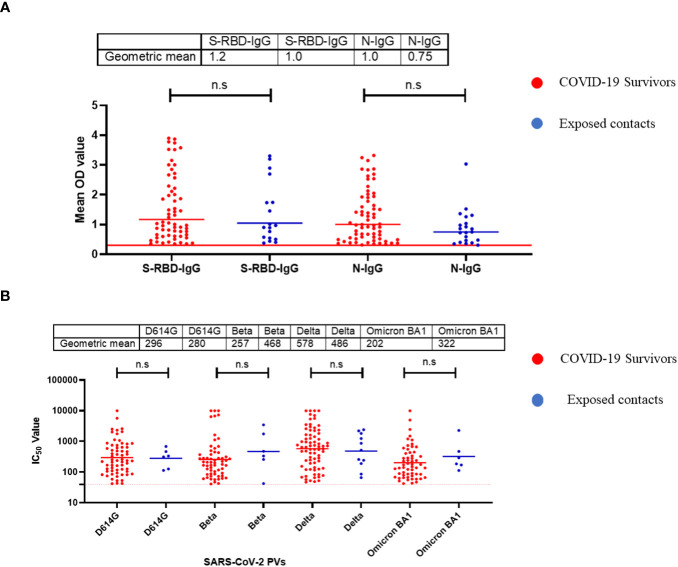
Binding antibody responses (IgG) to SARS-CoV-2 S-RBD and N proteins **(A)** and neutralizing antibody response to SARS-CoV-2 PVs **(B)** from COVID-19 survivors and their contacts in Southern Nigeria. Specific SARS-CoV-2 S-RBD and N antigen ELISA were used to measure the binding antibody response (IgG) from hospitalized COVID-19 convalescent survivors (89) and non-hospitalized asymptomatic contacts (34). We used a 1:100 dilution of the serum sample. Among those with binding antibody response, we selected some sera and measured the neutralizing titre (IC50) against SARS-CoV-2 PVs expressing the S-RBD of different variants (D614G, Beta, Delta and Omicron). The OD of the negative cutoff was selected as the mean multiplied by three standard deviations of three known negative samples (0.3) for binding antibody and (40) for neutralizing antibody response (limit of detection). The table shows the geometric mean at 95% CI. Statistical significance was calculated by Mann–Whitney test and p values are indicated. (Capped line with * indicating significance.

Using standardised protocols and validated kits (ReSARSCoV-2 (S-RBD and N) Kit (10180 and 10166, Zalgen Labs, LLC)), we estimated the binding antibody responses (IgG) of both hospitalized COVID-19 survivors and their non-hospitalized exposed contacts (exposed to SARS-CoV-2 positive individuals but with no history of COVID-19 symptoms or a positive SARS-CoV-2 polymerase chain reaction [PCR] test at the time of sample collection). This group included individuals who managed or provided care to COVID-19 acute or convalescent patients such as family members, clinicians, and nurses. Of the 123 sera tested (89 survivors and 34 contacts), both survivors and contacts had similar percentages of IgG responders to SARS-CoV-2 S-RBD and N protein (**Table 1**). Interestingly, the mean OD was not significantly different (Mann–Whitney test, p=0.55,0.09) between the survivors and the contacts both for S-RBD IgG and N-IgG (**Figure 1A**). From these, we selected those with binding antibodies and measured their neutralizing potential against four different SARS-CoV-2 pseudotype viruses (PV) expressing the full-length spike of the original Wuhan-Hu-1 isolate, and the successive variants- beta, delta and omicron BA1 respectively. Neutralizing antibodies against these selected SARS-CoV-2 PVs were similar to the binding antibody response data. Notably, both survivors and their contacts’ sera had neutralizing antibodies against one or more of the SARS-CoV-2 PVs, and no significant difference (Mann–Whitney test, p=0.94,0.23,0.81,0.25) was detected in the mean IC50 of both the survivor and contacts. (**Figure 1B**).

### Hybrid immunity and with different vaccines induced stronger binding and neutralizing IgG antibody responses to the SARS-CoV-2 RBD antigens.

**Figure 2:**
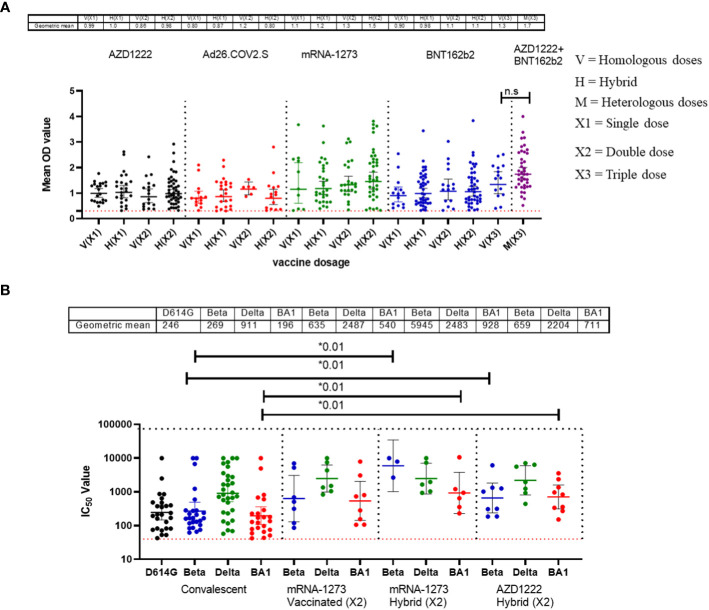
Binding antibody responses (IgG) to SARS-CoV-2 S-RBD proteins **(A)** and neutralizing antibody response to SARS-CoV-2 PVs **(B)** from COVID-19 vaccinees in Southern Nigeria. Specific SARS-CoV-2 S-RBD antigen ELISA was used to measure the binding antibody response (IgG) from COVID-19 vaccinees (521). We used a 1:100 dilution of the serum sample. Among those with binding antibody response, we selected some sera (50) and measured the neutralizing titre (IC50) against SARS-CoV-2 PVs expressing the S-RBD of different variants (D614G, Beta, Delta and Omicron). The OD of the negative cutoff was selected as the mean multiplied by three standard deviations of three known negative samples (0.3) for binding antibody and (40) for neutralizing antibody response (limit of detection). The table shows the geometric mean at 95% CI. Statistical significance was calculated by Mann–Whitney test and p values are indicated. (Capped line with * indicating significance).

Similar to the standardised protocols and validated kits described above, we estimated the binding and neutralizing antibody responses from COVID-19 vaccinated participants’ sera. We compared sera from different participants (n=517) that received four different vaccine types at different doses (AstraZeneca (ADV1222), Janssen (Ad26.COV2.S), Pfizer (BNT162b2) and Moderna (mRNA-1273). Not unexpectedly, we found that many vaccinated participants’ sera had positive binding antibody responses to both SARS-CoV-2 S-RBD and N antigens. The positive N IgG responses evidenced that vaccinated participants had prior exposure to either SARS-CoV-2 or cross-reactive coronaviruses as vaccines used were devoid of N antigens, thus providing evidence of previous infection and hybrid immunity. Interestingly, when we measured the binding antibody responses to S-RBD IgG in the SARS-CoV-2 vaccinated sera, participants with hybrid immunity and those that received different vaccine booster combinations (i.e. ADV1222 and BNT162b2) over three dosages had the strongest binding antibody IgG response to S-RBD (Mann–Whitney test, p=0.16) compared to those that received booster vaccinations with just one type of vaccines alone (**Figure 2A**). We further measured the neutralizing antibodies against the SARS-CoV-2 PV panels. Not unexpectedly, sera from vaccinated participants and those with hybrid immunity also had significantly stronger (Mann–Whitney test, p=0.01) neutralizing antibody responses to all the SARS-CoV-2 S-RBD PVs compared to the sera from convalescent participants (**Figure 2B**).

### Binding and neutralizing antibody responses from convalescent participants correlated with waves of COVID-19 in Southern Nigeria.

**Figure 3:**
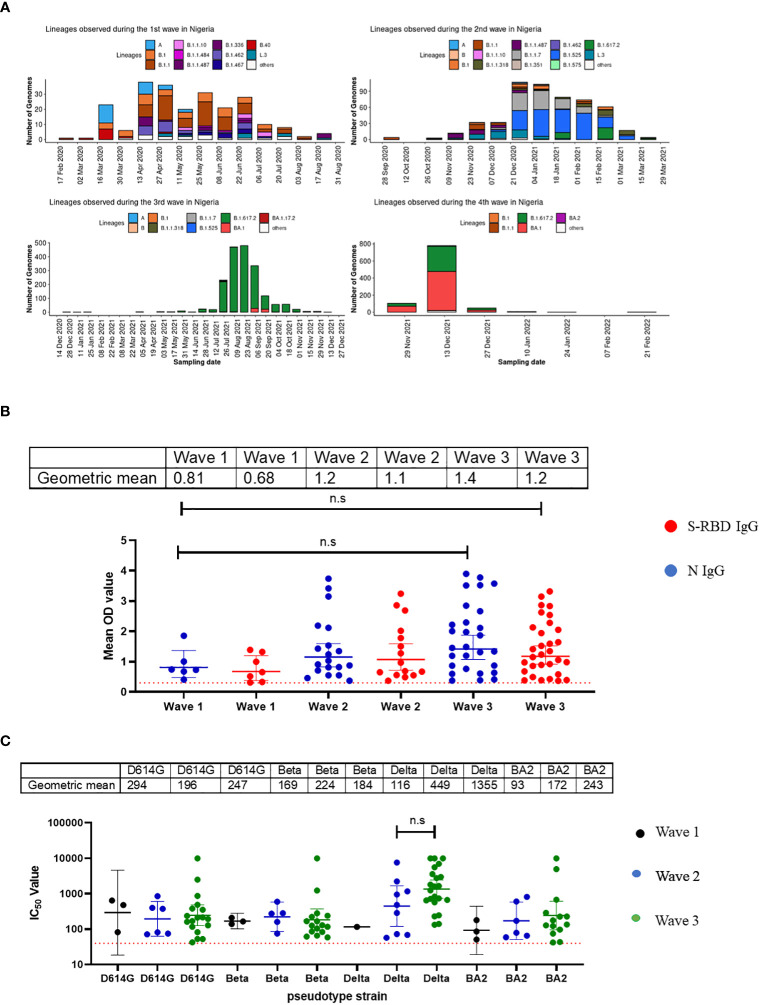
Binding antibody responses (IgG) to SARS-CoV-2 S-RBD and N proteins **(B)** and neutralizing antibody response to SARS-CoV-2 PVs **(C)** from COVID-19 survivors based on the waves of pandemic **(A)** in Southern Nigeria. Specific SARS-CoV-2 S-RBD and N antigen ELISA were used to measure the binding antibody response (IgG) from hospitalized COVID-19 convalescent survivors (89). The serum was grouped based on the time of infection/ diagnosis into the three waves of the COVID-19 pandemic. We used a 1:100 dilution of the serum sample. We also measured the neutralizing titre (IC50) against SARS-CoV-2 PVs expressing the S-RBD of different variants (D614G, Beta, Delta and Omicron). The OD of the negative cutoff was selected as the mean multiplied by three standard deviations of three known negative samples (0.3) for binding antibody and (40) for neutralizing antibody response (limit of detection ). The table shows the geometric mean at 95% CI. Statistical significance was calculated by Mann–Whitney test and p values are indicated. (Capped line with * indicating significance).

Nigeria experienced four major waves of COVID-19 to date. Wave one was from February 2020 to August 2020 and was dominated by the ancestral Wuhan strain (D614G), wave two from September 2020 to March 2021, was dominated by the eta and alpha variants, while the delta variant was the dominant wave three and lasted from April 2021 to November 2021 (13). Wave four, the omicron wave, began in December 2021 until today (**Figure 3A**). We evaluated the binding and neutralizing antibody responses from convalescent individuals’ sera collected during three of the four major waves of COVID-19 in Nigeria. By the fourth wave, samples were not prospectively collected due to extensive vaccine coverage, and relatively few people presented to the hospitals during the omicron wave. Samples collected were subdivided into different wave groups based on the date of COVID-19 diagnosis or hospital admission. Our results revealed that sera collected during the third wave, dominated by the delta variants, had the strongest binding antibody response. This was corroborated by our pseudotype-neutralizing antibody assay, which also showed that sera collected during the third wave had the strongest neutralizing antibody responses and the delta variant was the most frequently neutralized of the SARS-CoV-2 variants tested while the omicron was the least neutralized (**Figure 3B, C**).

### Pre-pandemic sera’s cross-reactive binding antibody responses to SARS-CoV-2 did not neutralize any of the SARS-CoV-2 or variants.

**Figure 4:**
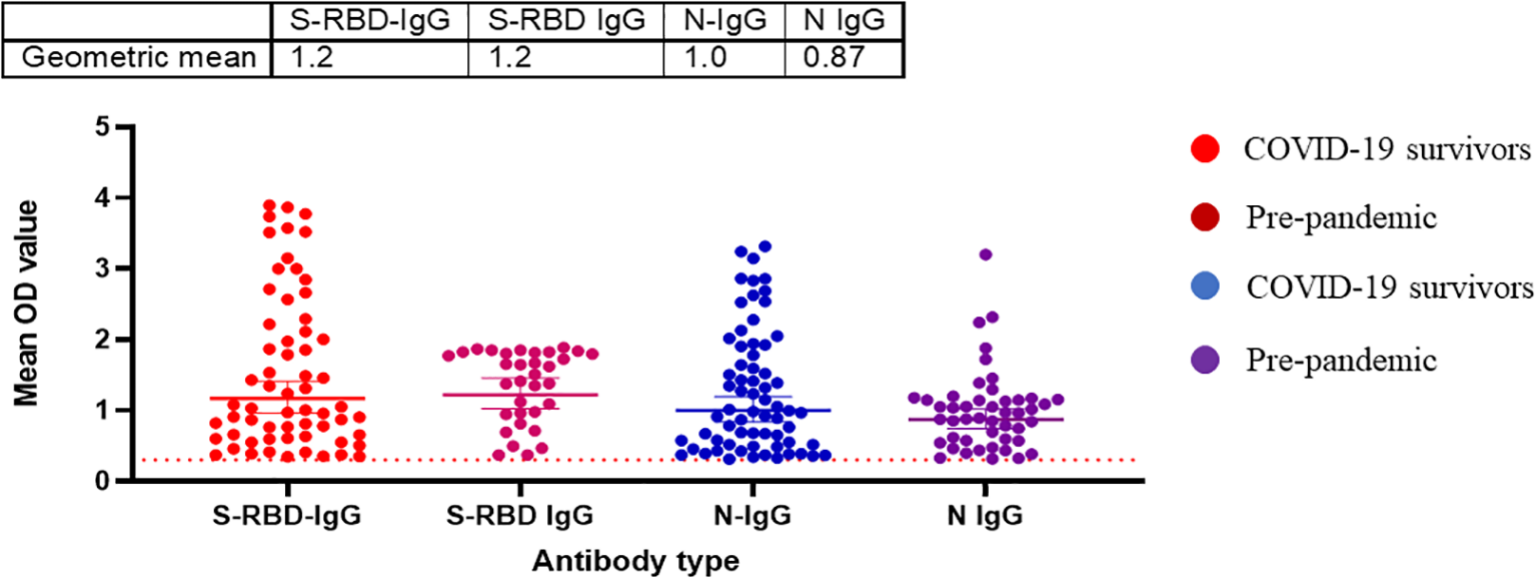
Pre-pandemic sera has detectable binding antibody responses (IgG) to SARS-CoV-2 S-RBD and N proteins similar to sera from COVID-19 survivors in Southern Nigeria. Specific SARS-CoV-2 S-RBD and N antigen ELISA were used to measure the binding antibody response (IgG) from sera (N=64) collected prior to the COVID-19 pandemic. We used a 1:100 dilution of the serum sample. The OD of the negative cutoff was selected as the mean multiplied by three standard deviations of three known negative samples (0.3).The table shows the geometric mean at 95% CI. Statistical significance was calculated by Mann–Whitney test and p values are indicated. (Capped line with * indicating significance).

Randomly selected sera (n=79) from positive and negative Lassa fever patients collected before the pandemic (2018-2019) were tested for binding antibody response to SARS-CoV-2 S-RBD and N antigens and neutralizing antibody responses to the same panel of SARS-CoV-2 pseudotype viruses (PVs) expressing the spike of the ancestral strain (D614G), beta, delta and omicron BA1. Notably, cross-reactive serological antibody responses to both S-RBD and N protein of SARS-CoV-2 were identified in 35(44%) and 47(60%) pre-pandemic sera available (**Table 1**). Interestingly, these responses were in the same range as binding antibody responses from documented COVID-19 survivors (**Figure 4**). However, none of the pre-pandemic sera had detectable neutralizing antibody responses to any SARS-CoV-2 S-RBD PVs in contrast to sera from COVID-19 survivors (Data not shown).

### Hospitalized COVID-19 survivors had T cell responses to both S and N proteins of SARS-CoV-2

**Figure 5:**
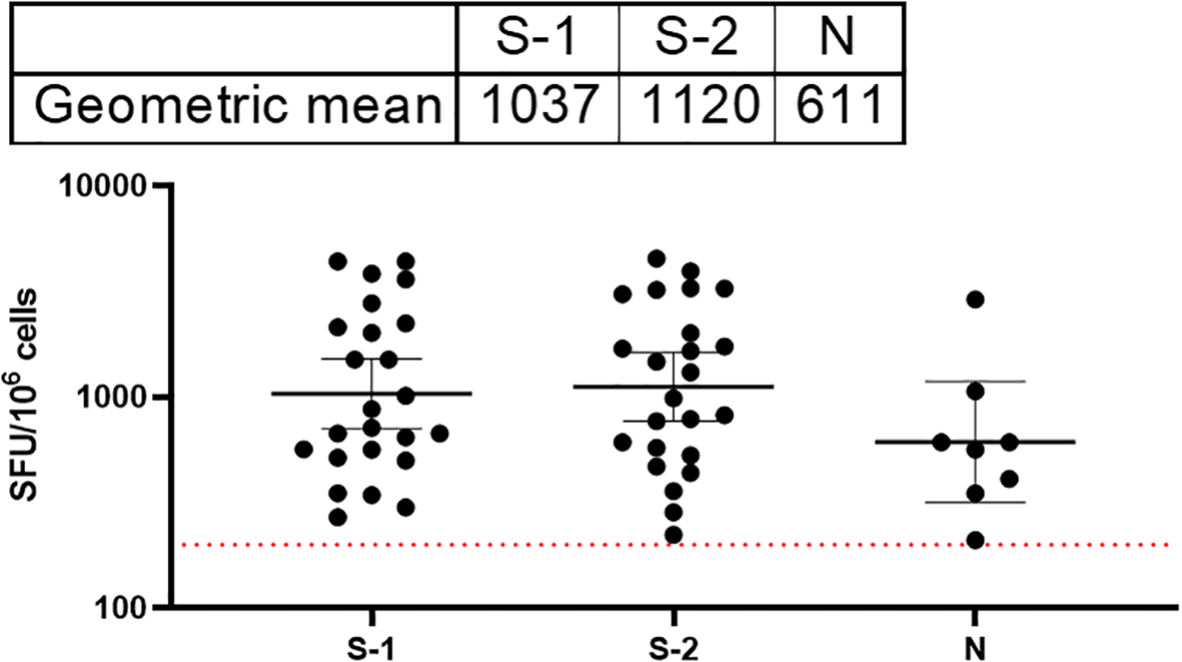
Stronger T cell responses to the SARS-CoV-2 spike proteins compared to the Nucleoprotein in COVID-19 survivors. The result (table showing geometric mean at 95% CI) showed stronger T cell response to both S1 and 2 compared to the N peptides. The average limit of detection (red dotted line=200) was calculated as the mean multiplied by one standard deviation of the three known negative samples.

T cell responses among COVID-19 survivors were evaluated using direct ex vivo interferon gamma (IFNγ) T cell ELISpot. SARS-CoV-2 spike (S1, S2) and N 15mer peptides from D614G strain were generated and used for this assay. Peptides were incubated with PBMCs from survivors (n=89) in culture overnight, and IFNγ spots were counted as a read-out for active T cell response (spot forming unit per 1 million cells). The average limit of detection (red line=40) was calculated as the mean multiplied by three standard deviations of the three known negative/naïve samples (individuals with no known previous exposure to SARS-CoV-2). Of the 89 samples analyzed for their T cell responses, 69% of the survivors were found to have T cell responses to either spike (S1, S2 peptides and N, or both peptides’ pools above detectable threshold for confirmed naive/negative individuals. The highest frequency of T cell response were observed in the S2 pool amino acid regions compared to the S1 and N peptides (**Figure 5**).

## Discussion

Details of the COVID-19 pandemic in Africa remain to be elucidated. The impact of SARS-Cov-2 was not as severe as in many other parts of the world (14, 15). For example, Nigeria, Africa’s most populous nation and 6^th^ most populous country in the world had less than one percent (0.01%) of the global COVID-19 morbidity and mortality (1). The factors responsible for the relatively low morbidity and mortality in Nigeria are not clear. The presence of uncharacterized endemic Coronaviruses or concurrent infections in Africa, have been postulated (16). Other factors such as differences in age distribution dynamics, testing have also been hypothesized as the drivers of the relatively low morbidity and mortality in Africa (2). As we enter the post-pandemic phase, opportunities are directed towards understanding such questions concerning natural and acquired immunity to SARS-CoV-2 in Africa and how it impacted different populations globally. Unfortunately, there is a paucity of information on the immunity to SARS-CoV-2 from the African continent as the majority of the COVID-19 research has come from resource rich and research intensive nations (17, 18). Here, we present our first insight into both natural and vaccine-acquired immune response in a small cohort of individuals in Southern Nigerians. Our cohorts include known survivors of COVID-19, vaccinated individuals, non-hospitalized asymptomatic but exposed individuals (contacts) and pre-pandemic samples.

Our data revealed a similar magnitude of binding and neutralizing antibody responses to both S-RBD and N antigens of SARS-CoV-2 from hospitalized survivors of COVID-19 and non-hospitalized asymptomatic survivors (**Figures 1A, B**). This corroborated previous seroprevalence data from both Nigeria and Ghana that identified high-binding antibody responses in asymptomatic individuals with no positive diagnosis of SARS-CoV-2 infection (19, 20). Here we extended these observations to demonstrate that sera from asymptomatic participants neutralized SARS-CoV-2 Spike PVs to the same level as sera from symptomatic COVID-19 survivors. This contrasted our observation in Lassa fever disease immunity where both hospitalized and non hospitalized patients generate binding antibody response to the Lassa virus GP and NP protein but neutralizing antibody response was mainly from the hospitalized patients (21). The binding antibody response to N antigens observed in both sera from symptomatic and asymptomatic individuals needs to be differentiated by peptide-based assays to determine if the antibody response is from SARS-CoV- related or other Coronaviruses. Interestingly, sera collected prior to the COVID-19 pandemic have binding antibody responses to both N and S-RBD antigens and none had neutralizing antibody response to any of SARS-CoV-2 Spike PVs, unlike sera from COVID-19 symptomatic and asymptomatic participants with both binding and neutralizing antibody responses to SARS-CoV-2 PVs. The presence of cross-reactive binding antibody responses but with no neutralizing function have been observed in pre-pandemic sera in other African countries such as SierraLeone and Uganda (16, 22). Future efforts to explore the non-neutralizing function of the pre-pandemic sera such as antibody dependent cytotoxicity (ADCC) and peptide arrays will help to determine the specificity and if pre-existing, cross-reactive bystander immunity may have played a role in reducing morbidity and mortality in Africans during the COVID-19 pandemic.

As expected, sera from individuals vaccinated with combinations of the different vaccines deployed in Nigeria demonstrated binding to S-RBD antigens and neutralizing antibody responses. Interestingly, sera from those with previous infection (either SARS-CoV-2 or seasonal coronaviruses) as well as vaccination (hybrid immunity) elicited stronger binding and broader neutralizing antibody responses compared to those with infection or vaccination alone (**Figures 2A, B**). Our data corroborated other studies that demonstrated the quality of hybrid immunity in contrast to either infection or vaccine induced immunity alone (23, 24). It will be interesting to determine whether the boosting immunity in documented hybrid immune cases is acquired by previous infection by SARS-CoV-2 variants or certain seasonal coronavirus. Interestingly Amanat et al. (2022) demonstrated that mice that were previously exposed to seasonal coronavirus had no boosting or inhibitory effects on subsequent vaccine immunity so-called immune imprinting (25). Given that much of the population have now been infected as well as vaccinated, future studies will be needed to elucidate the nature of prior coronavirus infection that caused this pre-pandemic ‘immune imprinting’ like phenomenon on subsequent SARS-CoV-2 immunity in Africans in an effort to understand why COVID-19 was less severe in a carefully documented populations in Africa.

**Figure 6:**
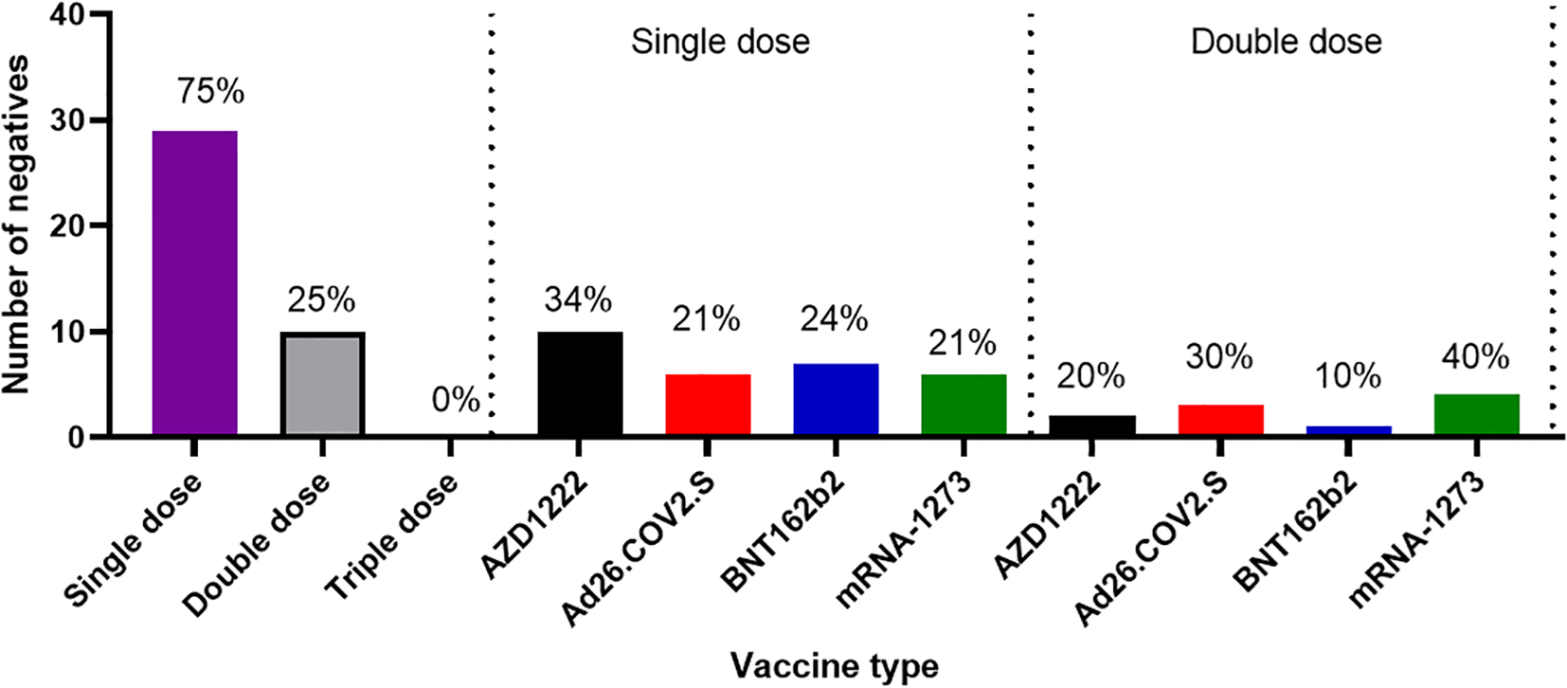
Cases of vaccine failures among COVID-19 vaccinees in Southern Nigeria. Vaccine failures were most frequent in individuals who only received a single dose of the vaccine compared to those with multiple booster immunizations, and no difference in cases of vaccine failure among those who received different types of vaccines.

Another outcome of this study was the observation of incremental binding antibody responses with frequency of vaccine boosters. Three doses of BioNTech/Pfizer vaccines elicited stronger binding antibody responses compared to the first and second vaccine doses (**Figure 2A**). However, for those that received three different vaccines, the heterologous vaccine prime and boost combinations resulted in stronger binding antibodies compared to homologous vaccine booster immunizations (Mann–Whitney test, p=0.16). Interestingly, the majority of vaccine failure (vaccinees with no binding antibody responses at the time of sampling) were most frequent in individuals that only received a single dose of the vaccine compared to those with multiple booster immunizations. More than two thirds’ sera with negative binding antibody responses from people documented to have been vaccinated, were in individuals with a single immunization, while one-third were in individuals that had received two immunizations, and none was found among those that have been immunized 3 or more times (**Figure 6**). No relationships were found in documented cases of vaccine failures or negative binding antibody responses between vector-based vaccines and mRNA-based vaccines both in the single and the double immunizations.

Nigeria experienced four major waves of COVID-19. Not surprisingly, our binding antibody and neutralizing antibody responses followed the waves of infection in Nigeria over time (**Figures 3A, B, C**). Not unexpectedly, (as has been previously reported in non-Africa nations) sera collected during the most recent wave was associated with the strongest binding and neutralizing antibody responses compared to those collected in the first and second wave. The third wave was dominated by the delta variant which was reflected in the titres of neutralizing antibodies to this variants corroborating similar studies in India showing stronger antibody response in the second wave dominated by the delta variants (26). The reason for the increase in antibody response post-delta wave may be due to the fact that delta variants infected more people than other variants in Nigeria. It could also be a function of boosting from prior infection or immunizations with repeated exposure and the resultant maturation of antibody affinity over time. Individuals in the third wave were likely to have had more exposure, or re-exposure to SARS-CoV-2 than second and first waves.

With regard to T -cell mediated immunity, our convalescent participants also had T cell responses to both S and N antigens. The strongest T cell response was observed against the highly conserved S-2 region of the Spike protein (**Figure 5**). Due to cold chain logistic and technical reasons, limitation of PBMC sampling made it difficult to have sufficient samples to draw further conclusions or to follow the kinetics of T cell response in vaccinated participants.

In summary, our data revealed that asymptomatic cases of COVID-19 generate similar magnitudes of both binding and neutralizing antibody responses to individuals with symptoms of COVID-19 in Nigeria. Notably, we were able to clearly separate the asymptomatic COVID-19 antibody responses from pre-existing antibody response by absence of neutralizing antibodies in cases in which we found to have a pre-pandemic pre-existing or cross-reactive immunity to SARS-CoV-2. We also showed that both vaccine and convalescent antibody responses were able to cross neutralize different circulating VOCs in Nigeria. In addition, convalescent antibody responses were found to correlate with the waves of SARS-CoV-2 in Nigeria. Lastly, hybrid immunity and heterologous vaccine boosting induced the strongest binding and broadly neutralizing antibody responses compared to vaccine or infection acquired immunity alone. This data is the first detailed study of SARS-CoV-2 immune responses acquired throughout the COVID-19 pandemic in Nigeria. Understanding the nature of the pre-existing cross-reactive SARS-CoV-2, non-neutralizing antibodies in African populations, and their possible impact on the relative COVID-19 disease resistance is a question that remains to be elucidated.

### Limitation of the current study

This study sampling was mainly from Southern Nigeria and sample sizes were variable for each study groups. Due to logistic and social security circumstances, we were unable to recruit participants from Northern Nigeria. Our data focused heavily on antibody immune responses. Due to lack of cold-chain resources we were unable to measure T cell response from immunized participants. Due to increased vaccination and population complacency, convalescent sera were largely unavailable during the 4^th^ wave of the pandemic.

## Data availability statement

The raw data supporting the conclusions of this article will be made available by the authors, without undue reservation.

## Ethics statement

The studies involving humans were approved by Nigerian National Health Research Ethics Committee and Redeemer’s University Institutional Review Board (SIP-NG-NHREC/01/01/2007-12/01/2021, ARISE-NHREC/01/01/2007-11/02/2022). The studies were conducted in accordance with the local legislation and institutional requirements. The participants provided their written informed consent to participate in this study.

## Author contributions

CU: Conceptualization, Data curation, Formal analysis, Funding acquisition, Investigation, Methodology, Project administration, Resources, Supervision, Validation, Visualization, Writing – original draft, Writing – review & editing. OA: Data curation, Investigation, Methodology, Project administration, Writing – review & editing. OJ: Data curation, Investigation, Methodology, Project administration, Writing – review & editing. BA: Data curation, Investigation, Methodology, Project administration, Writing – review & editing. IA: Data curation, Investigation, Methodology, Project administration, Writing – review & editing. OO: Data curation, Investigation, Project administration, Writing – review & editing. IB: Data curation, Investigation, Writing – review & editing. AC: Investigation, Methodology, Writing – review & editing. JC: Investigation, Methodology, Writing – review & editing. JA: Investigation, Methodology, Writing – review & editing. IB: Data curation, Visualization, Writing – review & editing. KA: Data curation, Formal analysis, Methodology, Project administration, Writing – review & editing. JO: Formal analysis, Project administration, Supervision, Writing – review & editing. PE: Methodology, Supervision, Writing – review & editing. PO: Investigation, Supervision, Validation, Writing – review & editing. AO: Investigation, Writing – review & editing. IA: Investigation, Writing – review & editing. VA: Investigation, Writing – review & editing. EB: Investigation, Writing – review & editing. OA: Investigation, Writing – review & editing. NA: Investigation, Project administration, Supervision, Validation, Writing – review & editing. EO: Investigation, Project administration, Supervision, Validation, Writing – review & editing. KU: Investigation, Project administration, Supervision, Writing – review & editing. SO: Investigation, Project administration, Writing – review & editing. OO: Investigation, Writing – review & editing. OK: Supervision, Validation, Writing – review & editing. ME: Investigation, Methodology, Writing – review & editing. VO: Investigation, Methodology, Writing – review & editing. OA: Investigation, Project administration, Supervision, Writing – review & editing. FE: Investigation, Writing – review & editing. MO: Investigation, Writing – review & editing. VI: Investigation, Writing – review & editing. OA: Investigation, Project administration, Supervision, Validation, Writing – review & editing. AC: Investigation, Project administration, Supervision, Writing – review & editing. SO: Investigation, Project administration, Supervision, Writing – review & editing. NO: Investigation, Methodology, Project administration, Writing – review & editing. SA: Investigation, Methodology, Project administration, Writing – review & editing. WI: Investigation, Methodology, Project administration, Writing – review & editing. MO: Investigation, Project administration, Supervision, Writing – review & editing. AA: Investigation, Project administration, Supervision, Writing – review & editing. AN: Data curation, Formal analysis, Methodology, Visualization, Writing – review & editing. EA: Data curation, Formal analysis, Methodology, Visualization, Writing – review & editing. GC: Data curation, Formal analysis, Methodology, Supervision, Visualization, Writing – review & editing. NK: Data curation, Formal analysis, Methodology, Writing – review & editing. AC: Formal analysis, Methodology, Supervision, Validation, Writing – review & editing. CG: Methodology, Supervision, Validation, Writing – review & editing. RK: Project administration, Supervision, Validation, Writing – review & editing. PT: Methodology, Project administration, Supervision, Validation, Writing – review & editing. NT: Methodology, Supervision, Validation, Visualization, Writing – review & editing. JH: Conceptualization, Funding acquisition, Project administration, Resources, Supervision, Validation, Visualization, Writing – review & editing. CH: Conceptualization, Funding acquisition, Project administration, Resources, Supervision, Validation, Visualization, Writing – review & editing.

## Author disclaimer

This work was supported by the Cambridge Africa Alborada COVID-19 Emergency fund grant nr. BB/R020116/1 and the Bill and Melinda Gates Foundation GIISER Project (ARISE- INV-036304). Under the grant conditions of the Foundation, a Creative Commons Attribution 4.0 Generic License has already been assigned to the Author Accepted Manuscript version that might arise from this submission. The findings and conclusions in this publication are those of the authors and do not necessarily reflect the positions or policies of the funders.
