# Supplementary material for: Immunological insights into COVID-19 in Southern Nigeria
Source: Front Immunol. 2024 Jan 23;15:1305586. doi: 10.3389/fimmu.2024.1305586 (PMC10844438; doi:10.3389/fimmu.2024.1305586)
Supplement: Supplementary file 1 [file Table_1.docx]

Supplementary Table 1 SARS-CoV-2 T cell ELISpot plate map

|  | 1 | 2 | 3 | 4 | 5 | 6 | 7 | 8 | 9 | 10 | 11 | 12 |
| --- | --- | --- | --- | --- | --- | --- | --- | --- | --- | --- | --- | --- |
| A | L10 | L10 | Anti-CD3 | Anti-CD3 | S-1 | S-1 | S-1 | S-2 | S-2 | S-2 | N-1 | N-1 |
| B | L10 | L10 | Anti-CD3 | Anti-CD3 | S-1 | S-1 | S-1 | S-2 | S-2 | S-2 | N-1 | N-1 |
| C | L10 | L10 | Anti-CD3 | Anti-CD3 | S-1 | S-1 | S-1 | S-2 | S-2 | S-2 | N-1 | N-1 |
| D | L10 | L10 | Anti-CD3 | Anti-CD3 | S-1 | S-1 | S-1 | S-2 | S-2 | S-2 | N-1 | N-1 |
| E | L10 | L10 | Anti-CD3 | Anti-CD3 | S-1 | S-1 | S-1 | S-2 | S-2 | S-2 | N-1 | N-1 |
| F | L10 | L10 | Anti-CD3 | Anti-CD3 | S-1 | S-1 | S-1 | S-2 | S-2 | S-2 | N-1 | N-1 |
| G | L10 | L10 | Anti-CD3 | Anti-CD3 | S-1 | S-1 | S-1 | S-2 | S-2 | S-2 | N-1 | N-1 |
| H | L10 | L10 | Anti-CD3 | Anti-CD3 | S-1 | S-1 | S-1 | S-2 | S-2 | S-2 | N-1 | N-1 |
|  |  | | | | | |  | | | | | |

Note

Each row represents a participant sample. While S-1 and S-2 were run in triplicates, the N antigen was run in duplicate.
